# A tunable microfluidic 3D stenosis model to study leukocyte-endothelial interactions in atherosclerosis

**DOI:** 10.1063/1.4993762

**Published:** 2018-01-02

**Authors:** Nishanth Venugopal Menon, Hui Min Tay, Kuin Tian Pang, Rinkoo Dalan, Siew Cheng Wong, Xiaomeng Wang, King Ho Holden Li, Han Wei Hou

**Affiliations:** 1 School of Mechanical and Aerospace Engineering, Nanyang Technological University, Singapore 639798; 2 Lee Kong Chian School of Medicine, Nanyang Technological University, Singapore 308232; 3 Department of Bioengineering, Imperial College, London SW72AZ, United Kingdom; 4 Institute of Molecular and Cell Biology, Agency for Science, Technology and Research (A*STAR), Singapore 138673; 5 Endocrinology Department, Tan Tock Seng Hospital, Singapore 308433; 6 Singapore Immunology Network, Agency for Science, Technology and Research, Singapore 138648; 7 Department of Cell Biology, Institute of Ophthalmology, University College, London EC1V9EL, United Kingdom; 8 Singapore Eye Research Institute, Singapore National Eye Centre, Singapore 168751

## Abstract

Atherosclerosis, a chronic inflammatory disorder characterized by endothelial dysfunction and blood vessel narrowing, is the leading cause of cardiovascular diseases including heart attack and stroke. Herein, we present a novel tunable microfluidic atherosclerosis model to study vascular inflammation and leukocyte-endothelial interactions in 3D vessel stenosis. Flow and shear stress profiles were characterized in pneumatic-controlled stenosis conditions (0%, 50% and 80% constriction) using fluid simulation and experimental beads perfusion. Due to non-uniform fluid flow at the 3D stenosis, distinct monocyte (THP-1) adhesion patterns on inflamed [tumor necrosis factor-α (TNF-α) treated] endothelium were observed, and there was a differential endothelial expression of intercellular adhesion molecule-1 (ICAM-1) at the constriction region. Whole blood perfusion studies also showed increased leukocyte interactions (cell rolling and adherence) at the stenosis of healthy and inflamed endothelium, clearly highlighting the importance of vascular inflammation, flow disturbance, and vessel geometry in recapitulating atherogenic microenvironment. To demonstrate inflammatory risk assessment using leukocytes as functional biomarkers, we perfused whole blood samples into the developed microdevices (80% constriction) and observed significant dose-dependent effects of leukocyte adhesion in healthy and inflamed (TNF-α treated) blood samples. Taken together, the 3D stenosis chip facilitates quantitative study of hemodynamics and leukocyte-endothelial interactions, and can be further developed into a point-of-care blood profiling device for atherosclerosis and other vascular diseases.

## INTRODUCTION

Atherosclerosis, the leading cause of cardiovascular diseases including myocardial infarction and stroke, is a chronic inflammatory disease widely associated with genetic predisposition and multiple risk factors such as hypertension, hyperlipidemia, and diabetes mellitus.^1^ It is marked by accumulation of cholesterol-containing low-density lipoproteins in the sub-endothelial space (intima), due to a complex interplay between activated leukocytes (monocytes, macrophages, and T cells) and the inflamed endothelium. Atherosclerosis is also a geometrically focal disease that preferentially affects vessel bifurcations, characterized by disturbed blood flow and low shear stresses (1–4 dyn/cm^2^).^2^ Several *in vitro* models have been developed to study lesion formation and disease progression, but these two-dimensional (2D) or three-dimensional (3D) co-culture models^3,4^ and cone-and-plate flow chambers,^5^ do not incorporate atherogenic vascular geometries and the associated complex flow profiles. While tubular *in vitro* models,^6^ tissue engineered vascular models,^7^ and animal models^8^ can accurately emulate the blood vessel physiology and hemodynamics, they are expensive and do not allow direct or long-term visualization.

Numerous microfluidic *in vitro* vascular models have been developed to study endothelial dysfunction, cancer metastasis, and angiogenesis or vasculogenesis.^9–11^ The ability to integrate multiple cell types and flow systems in microfabricated devices enables engineering of various atherogenic features including mechanical strain,^12^ monocyte phenotypes,^13^ disturbed flow,^14^ and platelet aggregation or thrombosis^15,16^ in microfluidic atherosclerosis models. However, reported stenotic microdevices are devoid of endothelial or blood components^15,17^ or have non-physiological 2D channel constrictions,^16,18,19^ thereby critically limiting studies on leukocyte-endothelial interactions at the atherosclerotic plaque. The recently reported “do-it-yourself” chip^20^ alleviates these issues by incorporating endothelial cells on a 3D stenosis, but the fabrication process is complicated and has limited control in stenosis geometry.

**FIG. 1.**
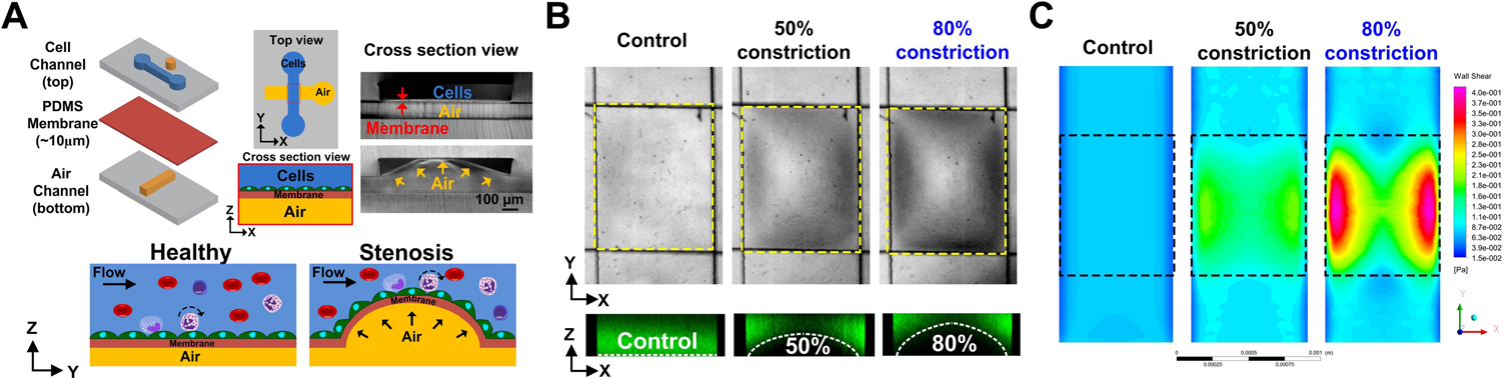
Characterization of the pneumatic-controlled 3D stenosis chip: (a) Schematic illustration of the multi-layered PDMS-based microfluidic device consisting of a cell culture channel (top) and an orthogonal air channel (bottom) separated by a thin (10 *μ*m) PDMS membrane. Air pumped into the bottom channel deflects the PDMS membrane upwards, thereby creating a 3D constriction (mimicking stenotic plaque) in the overlapping region (stenosis region) of the top channel. (b) (Top) Representative bright-field images showing different channel constrictions at the stenosis region (yellow dotted box). (Bottom) Confocal images of the cell channel loaded with FITC dye illustrating the corresponding channel constrictions (white dotted lines). (c) Fluid simulations highlighting the wall shear stress across the 3D constriction (dotted box). Distinct high shear (lateral sides) and low shear zones (apex) were observed for 50% and 80% constrictions.

Herein, we introduce a novel, pneumatically actuated 3D stenosis blood vessel model to study hemodynamics and leukocyte-endothelial interactions using microfluidics. The multi-layered polydimethylsiloxane (PDMS) microfluidic chip comprises of a cell culture channel (top) and an orthogonally placed air channel (bottom) separated by a thin PDMS membrane [Fig. 1(a)]. Air pumped into the bottom channel deflects the PDMS membrane upwards into the cell culture channel, thereby creating a tunable 3D constriction to mimic stenotic plaque of different severities. Fluid simulations were performed to study the flow and shear stress profiles at different channel constrictions. To understand the effects of flow disturbance in monocyte recruitment, THP-1 cells (monocytic cell line) were perfused over inflamed human umbilical vein endothelial cells (HUVECs) monolayer at different channel constrictions (50% and 80%) and wall shear stresses (1 and 10 dyn/cm^2^). We next characterized vascular inflammation in a perfusion culture, and our results indicated active endothelial cell alignment and differential flow-based inflammatory responses (ICAM-1) at the 3D bump in 80% channel constriction. Significant geometry-dependent effects in leukocyte adhesion were also observed under whole blood perfusion with a localized increase in monocytes binding at the apex of the 80% stenosis. Lastly, to demonstrate the potential of our 3D stenosis chip for inflammatory profiling, we perfused healthy and tumor necrosis factor-α (TNF-α) treated whole blood in 80% stenosis microchannels, and found a dose-dependent increase in leukocyte binding in inflamed blood samples. Taken together, the tunable-3D stenosis chip facilitates quantitative study of hemodynamics and shear-induced endothelial dysfunction on leukocyte-endothelial interactions. The model can be further developed into a point-of-care (POC) blood profiling device for diabetic and dyslipidimea patients to understand the risks associated with atherosclerosis plaque development.

## RESULTS

### Microfluidic device characterization

By regulating the air pressure into the bottom channel of the multi-layered PDMS device, 3D bump constrictions were formed at the overlapping region to recreate atherosclerosis plaques of different vessel constrictions [Fig. 1(b)]. To visualize the 3D stenosis region, we introduced fluorescein isothiocyanate (FITC) dye into the cell culture channel, and measured the fluorescence intensity linescan to determine the channel constriction in real-time (Fig. S1, supplementary material). Confocal images of FITC laden cell culture channel also confirmed the accuracy of our fluorescence method for measuring channel constrictions. Fluid simulations of the wall shear stress [Fig. 1(c)] and velocity (Fig. S2, supplementary material) indicated significant changes in flow profile from uniform laminar (control) to diverging flow patterns with increasing channel constrictions (50% and 80%), leading to the formation of distinct high shear (higher velocity) and low shear (lower velocity) zones around the stenosis. At 50% channel constrictions, shear stress (and velocity) magnitudes were higher at the stenosis in comparison to its apex, while for 80% constrictions, ∼4-fold increase in wall shear stress was observed at the lateral sides in comparison to the centre region (apex). This change in flow profile was due to the increased lumen irregularity at 80% constriction that led to increased flow through the lateral sides of the stenosis and minimal flow through the centre. As expected, the simulated flow profiles at the stenosis corroborated with the experimental flow profile of 10 *μ*m beads (1 dyn/cm^2^) (Fig. S3, supplementary material). For the control (no constriction) channel, beads distribution was relatively uniform with similar flow velocities across the channel width (x-axis). At 50% constriction, more beads were flowing along the sides at higher flow velocities due to the increased fluid flow. Negligible beads were flowing through the apex region (narrowest) at 80% constriction, and the bead flow velocities at the side channels were the highest among all conditions tested.

### Monocyte-endothelial cell interaction

**FIG. 2.**
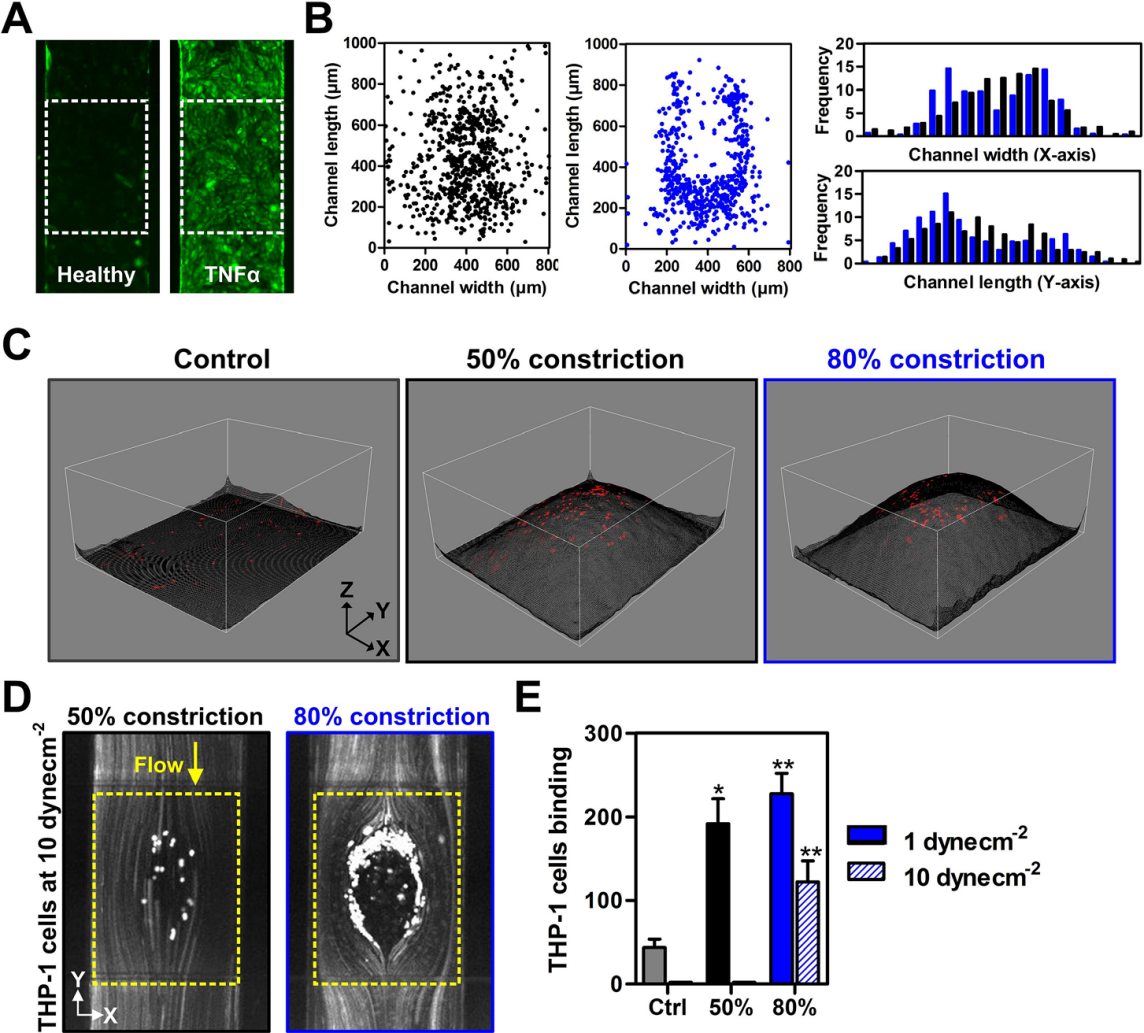
Monocyte-endothelial interactions at the stenosis region under flow: (a) Fluorescence images of the ICAM-1 expression (green) of healthy and TNF-α treated endothelial (HUVECs) monolayer. (b) Scatter and distribution plots indicating distinct adherence patterns of THP-1 on TNF-α treated HUVECs over 50% (black) and 80% (blue) stenosis at 1 dyn/cm^2^. (c) 3D image reconstruction of stenosis regions generated from FITC fluorescence distribution (see methods). Overlaid red dots indicate THP-1 adhesion on the 3D bump. (d) Stacked fluorescence images of THP-1 adherence to 50% and 80% stenosis (yellow dotted box) at 10 dyn/cm^2^. (e) THP-1 binding efficiencies to HUVECs at different channel constrictions and flow conditions (n = 3). Data are presented as mean ± s.d. (*p< 0.05, ** p < 0.01).

HUVECs were seeded in the cell channel (top) and cultured for 24 h to form a confluent monolayer on the PDMS membrane. Prior experiments, HUVECs were treated with TNF-α (10 ng/ml) for 24 h to induce vascular inflammation and upregulation of ICAM-1 expression, a key leukocyte adhesion marker [Fig. 2(a)]. Besides ICAM-1, vascular cell adhesion molecule-1 (VCAM-1) is also reported to play a prominent role in monocyte adhesion^21^ and progression of atherosclerosis.^22^ In our experiments, although an increase in both ICAM-1 and VCAM-1 expression in HUVECs during inflammation was detected, VCAM-1 expression was significantly lower than ICAM-1, making it difficult to visualize and quantify reliably (Fig. S4, supplementary material). Hence, ICAM-1 was used as a representative vascular inflammatory marker for subsequent experiments. THP-1 cells (stained with Cell Tracker™ green dye) were perfused over the inflamed HUVECs at 1 dyn/cm^2^ (atherogenic shear stress ∼1–4 dyn/cm^2^). As shown in Fig. 2(b), there was minimal monocyte adherence in the control chip (no constriction) while significant monocytes were bound to HUVECs in the constricted channels. Distinct distribution patterns of THP-1 adherence were also observed for 50% and 80% constriction channels, with higher cell densities at the centre of the 50% constriction channel, while majority of cell adhesion occurred at the proximal and lateral sides in the 80% constriction [Fig. 2(b)]. These differences were consistent with the non-uniform flow profiles obtained from the fluid simulations and beads perfusion (Figs. S3 and S5, supplementary material). A 3D image reconstruction of the stenosis region overlaid with THP-1 adherence pattern clearly indicated absence of THP-1 binding at the apex (narrowest ∼20 *μ*m gap height) of the bump in 80% channel constriction due to THP-1 cell size (∼20 *μ*m) which indicates an experimental artefact due to the small channel dimensions [Fig. 2(c)]. To characterize THP-1 adhesion on inflamed HUVECs at higher atheroprotective shear stresses (>10 dyn/cm^2^),^18^ we increased the wall shear stress to 10 dyn/cm^2^ and cell binding was completely eliminated in the 50% constriction channel [Fig. 2(d)]. THP-1 adhesion at 80% stenosis was also significantly reduced as compared to 1 dyn/cm^2^, but the distribution pattern remained similar [Fig. 2(e)]. These results clearly demonstrate the importance of vessel constriction (or plaque size) and resultant flow profile in regulating monocyte-endothelial interactions in the progression of atherosclerosis.

### Differential vascular inflammation during perfusion culture

**FIG. 3.**
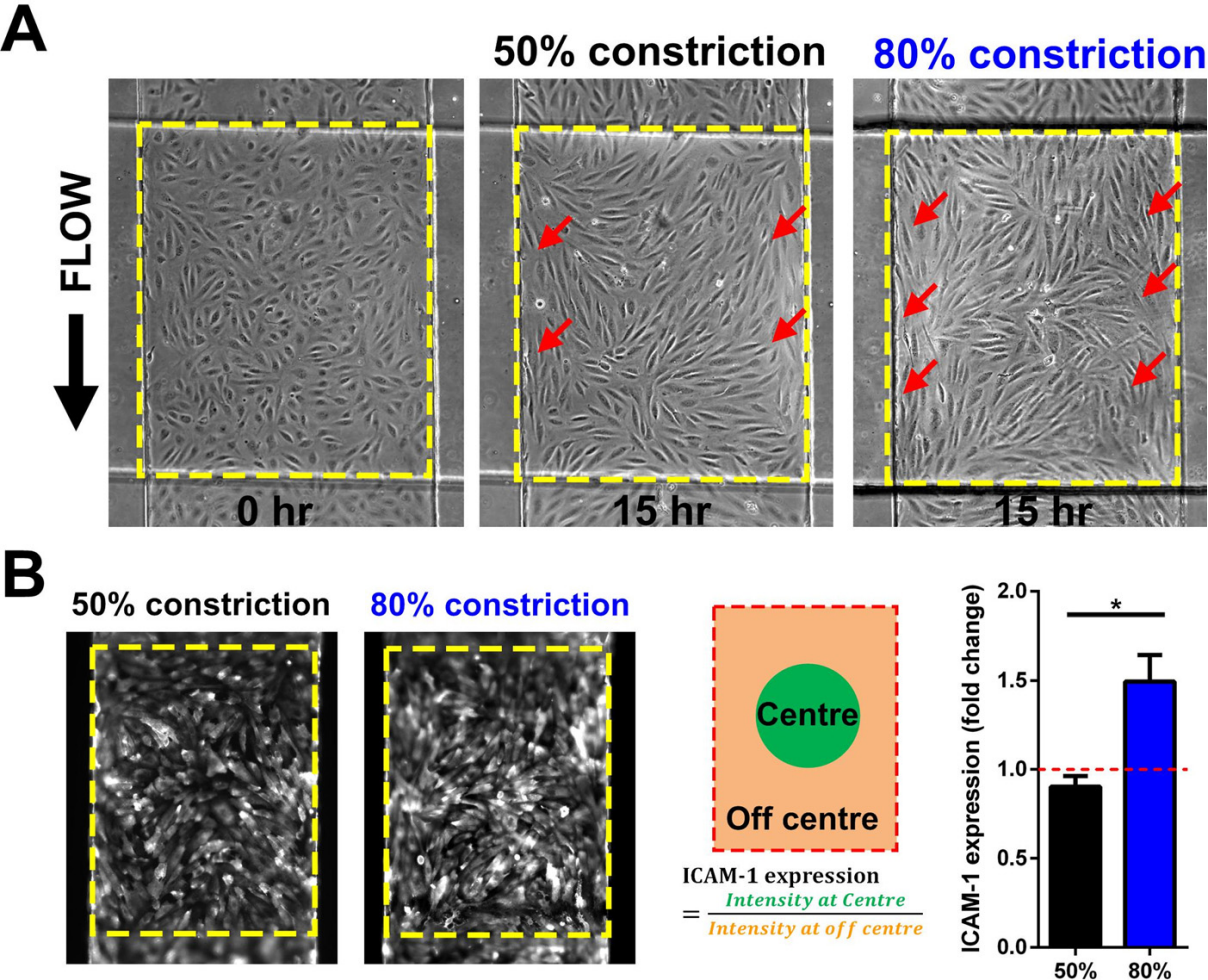
Shear-induced vascular inflammation under perfusion culture: (a) Phase contrast images of TNF-α treated HUVECs monolayer at different constrictions after 15 h of perfusion culture (1 dyn/cm^2^). Active cell alignment parallel to the flow was observed for HUVECs along the high shear zones (red arrows) for 50% and 80% constrictions. (b) Representative fluorescence images of ICAM-1 expression on HUVECs at the stenosis region (dotted box). Higher localized ICAM-1 expression was observed at the centre of the 80% constriction as compared to surrounding bump region (n = 3). Data are presented as mean ± s.d. (*p< 0.05).

To study shear stress-dependent endothelial activation, cell culture media containing TNF-α (10 ng/ml) were perfused over HUVECs in the cell culture channel at 1 dyn/cm^2^ for 15 h. The effects of non-uniform flow profile over the 3D stenosis were evident as HUVECs mostly aligned parallel to the flow direction on the lateral sides of the stenosis (higher shear), while cells remained randomly orientated at the centre of the stenosis, as well as the pre- and post-constriction areas of the chip [Figs. 3(a) and S6 (supplementary material)]. In addition to the distinct morphological changes, ICAM-1 endothelial expression was 4 times higher with TNF-α treatment as compared to control (untreated), and there were negligible differences between TNF-α-treated static and perfusion culture, which was consistent with prior observations^23^ (Fig. S6, supplementary material). Average ICAM-1 expression was also similar at the 3D stenosis, pre- and post-stenosis regions in both 50% and 80% constrictions (Fig. S6, supplementary material). Interestingly, we noticed a heterogeneous ICAM-1 expression at the 80% stenosis region, where ICAM-1 expression at the low shear zone (apex) of the 80% constriction was 50% higher (P < 0.05) in comparison to the lateral and the proximal-distal sides of the stenosis, an effect which was not observed at the 50% stenosis [Fig. 3(b)]. This differential inflammation may be attributed to the low shear stress at the apex^24^ and the mechanical strain^25^ experienced by the cells during membrane deflection.

### Leukocyte-endothelial interactions under whole blood perfusion

**FIG. 4.**
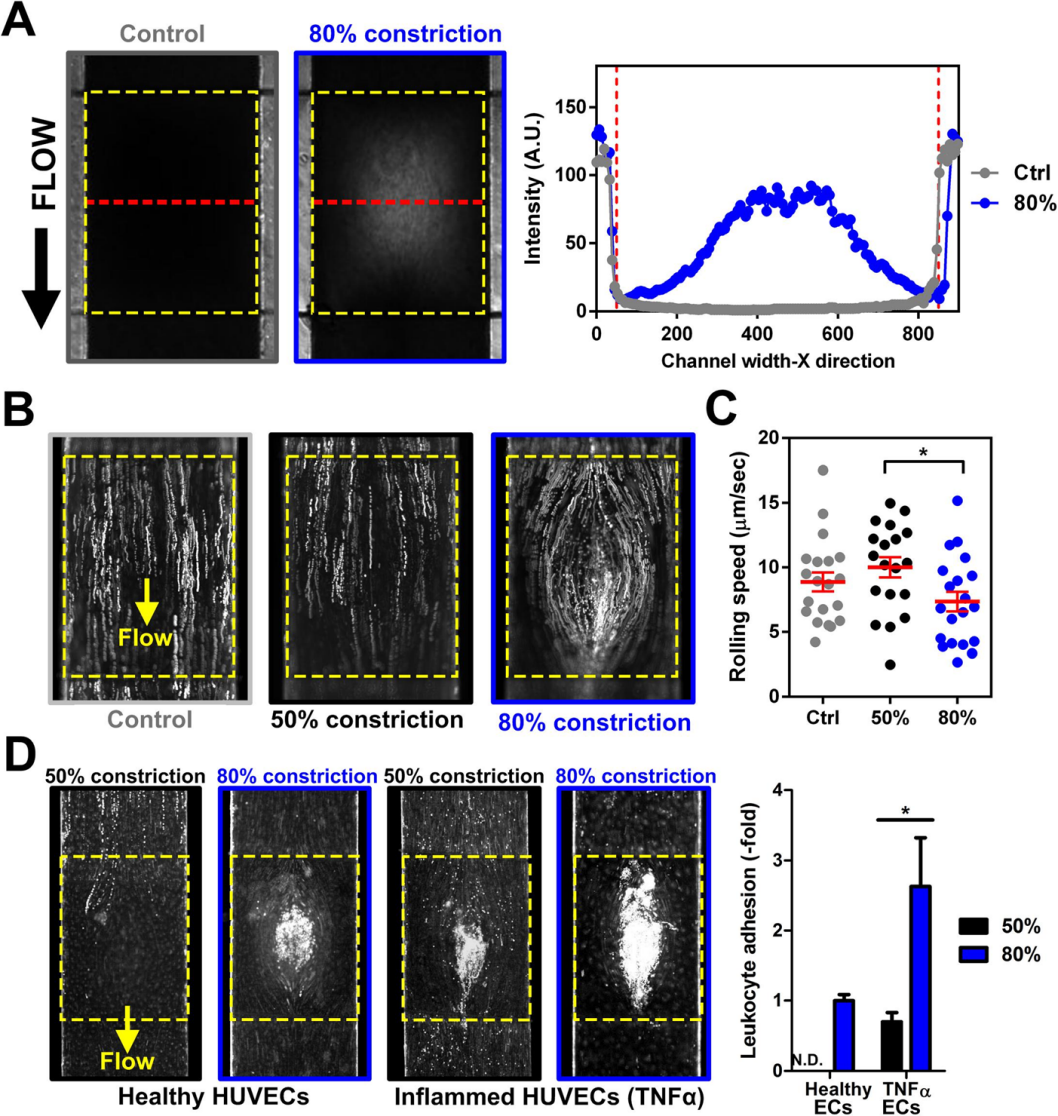
Whole blood perfusion in the developed stenosis device: (a) Bright-field images and intensity linescans (measured along red dotted lines) illustrating distinct differences in whole blood flow over the stenosis region (yellow dotted box). (b) Overlaid fluorescence images indicating differential leukocyte (R6G-stained) rolling trajectories on TNF-α treated HUVECs along the stenosis region. (c) Leukocyte rolling speed at different constrictions (n = 3). (d) Leukocyte adhesion at the stenosis region after 4 h of whole blood perfusion (1 dyn/cm^2^) over healthy and TNF-α treated HUVECs at different channel constrictions (n = 3). A significant increase in leukocyte adhesion was observed on the inflamed HUVECs at 80% constriction. Data are presented as mean ± s.d. (*p < 0.05).

After studying the influence of shear stresses on THP-1 adhesion and endothelial inflammation on the 3D stenosis, we performed whole blood experiments to characterize leukocyte interactions in our 3D stenosis model. Instead of using FITC intensity linescan, channel constrictions under blood perfusion were determined with bright-field intensity linescans, whereby decreased blood flow over the stenosis region resulted in higher intensities [Fig. 4(a)]. Citrated human whole blood (healthy) samples, stained with Rhodamine 6G (R6G) for platelets and leukocytes identification, were perfused into the inflamed (TNF-α treated) endothelial cell culture channels at 1 dyn/cm^2^. Time-lapse videos (0.5 s interval for 1 min) were captured at the stenosis region to quantify leukocyte rolling trajectories and velocities. As shown in Fig. 4(b), leukocyte rolling trajectories in control (no constriction) and 50% constriction channels were linear along the flow direction based on the stacked fluorescence images. However, leukocyte rolling at 80% constriction was curvilinear, which coincided with simulated and bead flow profiles around the centre bump region. Average leukocyte rolling velocities across the centre of the 80% constriction were also significantly lower as compared to 50% constriction or the control channels, which could be attributed to the prominent low shear zone at the centre for 80% stenosis [Fig. 4(c)]. Next, we perfused healthy whole blood over healthy and TNF-α treated HUVECs for 4 h at 1 dyn/cm^2^ to investigate the impact of vessel narrowing and vascular inflammation in leukocyte adherence [Fig. 4(d)]. For healthy endothelial monolayer, there was negligible leukocyte binding in control (data not shown) and 50% constriction regions, while significant leukocyte adherence was observed at the centre of 80% stenosis. For inflamed endothelium, increased leukocyte adhesion was evident with higher leukocyte adherence (∼3-fold) at 80% stenosis than 50% stenosis. To our surprise, the leukocyte adhesion pattern was different from earlier observations with THP-1 cells, and was likely due to smaller leukocyte sizes (∼10–15 *μ*m as compared to THP-1, ∼20 *μ*m) and the high red blood cells (RBCs) content. Finally, platelet adhesion was present in the pre- and post-constrictions sections at 80% stenosis (Fig. S7, supplementary material), and was in agreement with previous work reporting shear-induced platelet aggregation.^26^

### Whole blood profiling based on leukocyte adhesion

**FIG. 5.**
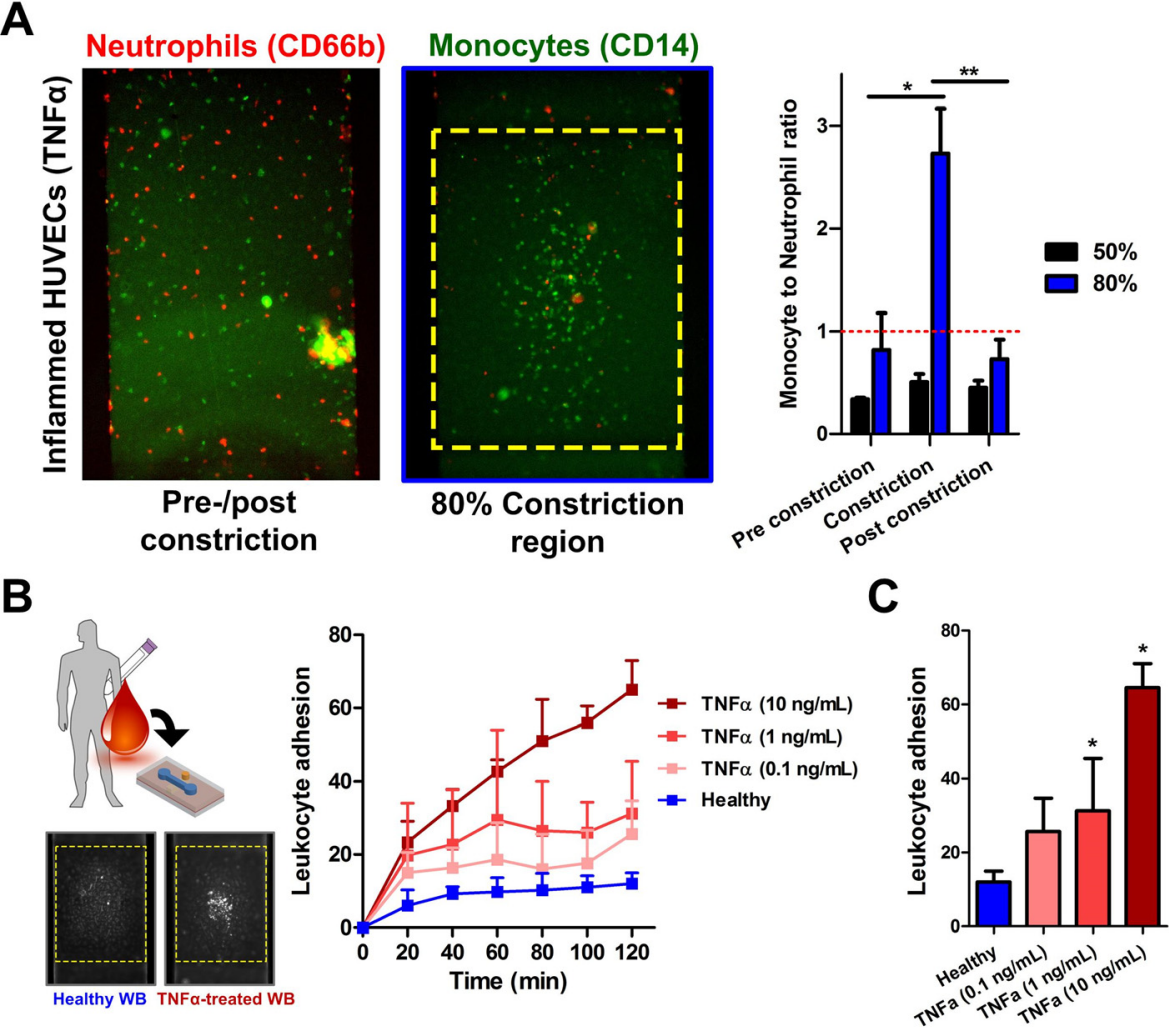
Leukocyte-endothelial profiling in 80% stenosis microchannel: (a) Leukocyte subtype characterization on inflamed HUVECs along the vascularized channel at different constrictions. A significant increase in monocytes (CD14+) adhesion over neutrophils (CD66b+) was observed at 80% stenosis region as compared to pre- and post-constriction (n = 4). (b) Differential leukocyte adhesion at the stenosis of healthy HUVECs over time (1 dyn/cm^2^) using whole blood inflamed with different TNF-α concentrations. Inset fluorescence images indicating leukocyte binding mostly at the apex region for inflamed blood. (c) Dose-dependent increase in leukocyte adhesion for inflamed blood after 2 h of perfusion (n = 4). Data are presented as mean ± s.d. (**p < 0.01, *p < 0.05).

To further understand the leukocyte-endothelial interactions, we perfused unstained whole blood (1 dyn/cm^2^ for 2 h) over inflamed HUVECs at 80% stenosis, and subsequently stained for neutrophils (CD66b+) and monocytes (CD14+). For 50% constriction channel, there were twice as many neutrophils adhered to the endothelium as compared to monocytes (monocytes to neutrophils ratio ∼0.5) along the entire device which was expected since neutrophils are the most abundant leukocytes in the blood. Interestingly, we observed a significant increase (∼3-fold) in monocyte count at the 80% stenosis region but not other parts of the channel [Fig. 5(a)]. This result was similar to previous observations on monocyte recruitment in areas of low shear^27^ and further highlights the physiological relevance of our developed model to study monocyte pathogenesis in atherosclerosis. Finally, we demonstrated the potential application of our atherosclerosis bio-chip in inflammatory profiling based on leukocytes adhesion under whole blood perfusion. Healthy blood and TNF-α treated (10 ng/ml, 1 ng/ml, and 0.1 ng/ml for 2 h) blood samples were introduced (1 dyn/cm^2^) over healthy endothelial monolayer maintained at 80% constriction. TNF-α is known to affect leukocyte functions,^28^ and also resulted in upregulation of endothelial E-selectin, VCAM-1, and ICAM-1 expression in our model within 2 h (Fig. S8, supplementary material). While there were negligible leukocytes binding observed for healthy blood over time, a dose-dependent increase in leukocyte adhesion was observed for TNF-α treated blood over 2 h [Fig. 5(b)] with an average increase of ∼2 to 10-fold in leukocyte adhesion [Fig. 5(c)]. Taken together, these results suggest leukocyte adhesion as an interesting and novel functional biomarker for POC immune health profiling using liquid biopsy.

## DISCUSSION

There are several risk factors well-associated with atherosclerosis (smoking, high blood cholesterol, high blood glucose, hypertension, etc.), and it is known that endothelial dysfunction plays a pivotal role in the early stage development of atherosclerotic lesions.^29^ Blood vessel stenosis narrows blood vessel lumen due to increased lipid deposition and foam cell formation, which can result in disturbed blood flow, shear-dependent endothelial dysfunction, and enhanced leukocyte recruitment. In this study, we introduced a stenosis model to study the effects of flow disturbance on vascular inflammation and leukocytes interactions in vessel stenosis. In contrast to existing 3D microfluidic constriction models requiring complex micromachining,^15,30^ we use standard microfabrication and pneumatically controlled PDMS membrane deflections to create tunable physiological relevant 3D plaque geometries to mirror the severity of vessel constriction in atherosclerosis. It should be noted that tunability refers to the ability to create different channel constrictions by pneumatic actuation of the membrane. This asymmetrical stenosis geometry is commonly used in *in vitro*^6,31^ and *in vivo*^26^ atherosclerosis models to study the hemodynamics effects in early stage atherosclerosis. Compared to 2D stenosis models whereby the channel constriction is at the sidewall,^16^ our studies have several key differences in flow disturbance due to the 3D constrictions. First, our channel constriction is along the channel bottom which provides sufficient imaging area for real-time monitoring and quantification of leukocyte-endothelial interactions. At lower occlusion severity (50% channel constriction), the disturbed flow profile in our model is similar to prior observations with high shear stress at the stenosis region.^6^ Second, an increase to 80% channel constriction results in the formation of low shear regions at the apex of stenosis. This is confirmed by fluid simulations and experimental studies, and can be related to a bean-shaped lumen with a narrow centre (apex) and broader lateral sections *in vivo.*^32^ As future work, we can increase the channel dimensions (x and z-axes) and length of constriction (y-axis) to give a more uniform decrease in cross-sectional (lumen) area. This will also help to minimize experimental artefacts as the constricted channels are larger than immune cells, and is more consistent with *in vivo* studies on leukocyte-endothelial interactions.

ICAM-1 is a mechano-sensitive inflammatory adhesion protein and is upregulated with increased shear stress (∼8.6 to 20 dyn/cm^2^).^33–35^ In our model, no change in ICAM-1 expression was observed between static and perfusion culture and across different sections of the stenosis chips. We hypothesize that this is due to the low shear stress (1 dyn/cm^2^ for 12 h) used in our studies which is in agreement with previous observations by Tsou *et al.*^36^ Second, the simultaneous mechanical (shear) and chemical (TNF-α) stimuli on endothelial cells might also lead to insignificant differences in ICAM-1 expression.^37^ Interestingly, ICAM-1 expression was increased at the apex region (low-shear) of the 80% channel constriction. One possible explanation is the mechanical strain exerted on the endothelial cells due to *in situ* stretching of the PDMS membrane. As endothelial ICAM-1 expression can be modulated by mechanical strain,^25,38^ future work is warranted to investigate the endothelial dysfunction in well-defined mechanical and flow-based atherogenic microenvironment.

In the whole blood perfusion studies, we observed key leukocyte recruitment processes including cell rolling and adherence to inflamed endothelium. Interestingly, while neutrophils binding was dominant elsewhere, we saw a significant increase in monocyte adherence at the low-shear centre region of the 80% stenosis. This is in good agreement with previous reports that have highlighted on the role of monocyte adhesion as a primary driver for atherosclerosis progression *in vivo.*^39^ Such lesions increase endothelial barrier permeability that will accelerate lipid deposition and plaque growth, and its eventual rupture resulting in thrombosis or acute ischemia.^40^ Major limitations in this study are the laminar flow profile and the small channel dimensions which may not fully recapitulate the flow complexities and mechanical stimulus in atherogenic arteries. Future work includes the use of a pneumatic pump to introduce complex flow profiles (pulsatile flow, oscillatory flow) to study atherogenic conditions. While our cell-based model enables real-time visualization of leukocyte-endothelial interactions under whole blood perfusion, it does not fully mimic the atherosclerotic microenvironment (absence of perivascular cells and humoral environment) for more complex processes including cell-matrix interactions and foam cell formation. Nevertheless, this pneumatic-controlled microdevice provides a simple and robust method to study flow-induced vascular inflammation and whole blood perfusion in different stenotic conditions which are important in the early stages of atherosclerosis development.

Finally, as a proof-of-concept for clinical testing, we applied our model to characterize leukocyte adhesion in healthy and inflamed blood, which could serve as a potential POC blood profiling tool for atherosclerosis risk assessment. We were able to differentiate healthy and TNF-α-treated (0.1 to 10 ng/ml) blood samples in a dose-dependent manner based on leukocyte adhesion at 80% stenosis. Although these TNF-α concentrations are higher than reported physiological levels (∼0.02 ng/ml),^41,42^ these data strongly suggest the potential of leukocyte adhesion as a functional biomarker for monitoring leukocyte-endothelial interactions in stenotic microchannels For future POC testing, we can coat recombinant inflammatory adhesion proteins (ICAM-1, VCAM-1, and E-selectin)^43^ in our devices instead of endothelial cells for more robust performance. In addition, we intend to screen blood samples obtained from patients suffering from inflammatory disorders such as diabetes or dyslipidimea to assess the clinical efficacy for leukocyte profiling and cardiovascular risk assessment.^13^

In summary, we have developed a novel blood vessel model with tunable 3D stenosis to mimic atherosclerotic plaque. We demonstrated significant changes in flow profiles at the stenosis region of different channel constrictions, and studied its impact on vascular inflammation and leukocyte interactions under whole blood perfusion. As proof-of-concept for POC testing, we applied the model to characterize leukocyte adhesion density using liquid biopsy, which can be further developed into a biomarker for atherosclerosis risk assessment in cardiovascular disorders.

## METHODS

### Microdevice fabrication

The multi-layered PDMS (Dow Corning) microfluidic device consists of a top cell culture channel (H × W of 100 *μ*m × 800 *μ*m), a bottom air channel (H × W of 100 *μ*m × 1000 *μ*m) separated by a 10 *μ*m PDMS membrane. SU-8 (Microchem) based photolithography was performed on silicon (Si) wafer to obtain the positive relief of the channel designs following which soft lithography was used to transfer the design on to PDMS. Briefly, PDMS prepolymer was mixed in the ratio of 10:1 (w/w) with curing agent and poured over a patterned Si wafer. The mixture was degassed and cured for 2 h at 80 °C, before peeling it off the wafer carefully. A biopsy puncher (1.5 mm) was used to define the inlet and outlet in the top cell culture channel. An additional hole was punched beside the channel on the top PDMS layer to serve as the inlet for the bottom channel. For PDMS membrane fabrication, uncured PDMS was poured onto a clean and silanized (Trichloro Silane, Thermo Fisher Scientific) Si wafer, spin coated (Speciality Coating Systems) at an rpm of 2400 for 5 min and maintained at 80 °C for 2 h (PDMS curing) to achieve a 10 *μ*m thick membrane. The device assembly was performed in two stages. First, the cell culture channel was plasma bonded (Harrick Plasma Cleaner) to the spin coated Si wafer with the PDMS membrane, followed by carefully cutting out this layer with the PDMS membrane. The second step involved bonding the top PDMS layer (with the membrane) to the bottom layer of PDMS. The two layers were plasma treated and aligned orthogonally before bonding.

### Device characterization

FITC dye (1 *μ*M, Sigma-Aldrich) with Endothelial cell Growth Media (EGM-2, Lonza) was introduced into the cell culture channel and imaged using a fluorescence microscope (Nikon Eclipse Ti). Channel constrictions were realised by pumping-in air into the pneumatic channel using a syringe pump (Chemyx, Inc.) while simultaneously monitoring the FITC fluorescence-linescan intensity. The fluorescence linescan intensity drops as the channel constriction increases, which was used to measure the percentage of channel constriction (Fig. S1, supplementary material). A 3D reconstruction of the stenosis region was performed from the FITC fluorescence images (at different channel constrictions) using ImageJ (NIH) for better visualization of the 3D-stenosis. Confocal imaging (LSM 800, Carl Zeiss) of the FITC-loaded channels was performed to visualize the 3D stenosis. Flow characterization was performed by pumping 10 *μ*m fluorescent polystyrene beads (Bangs Laboratories) through the culture channel. Time-lapse images were obtained to calculate the beads rolling velocities which were processed using ImageJ.

### Fluid simulation studies

3D microfluidic model was constructed using Solidworks 2015 (Dassault Systèmes SolidWorks Corp.) and imported to ANSYS Workbench 17 (ANSYS, Inc.) for geometry discretization and numerical simulation using ANSYS FLUENT. Inlet flow velocity was set at 10 *μ*l/min, which corresponded to ∼1 dyn/cm^2^. Non-slip boundary conditions were applied to all channel walls, and outlet was defined as a pressure outlet with atmospheric pressure. The Navier-Stokes equations were solved using 2nd order accuracy. The Semi-Implicit Method for Pressure-Linked Equations (SIMPLE) scheme was used for pressure-velocity decoupling. All simulations were conducted by using an iterative and segregated solution method. A residual sum for continuity and momentum of 1 × 10^−6^ was set as a convergence criterion. The working fluid was assumed to be water (homogeneous, single phase, Newtonian fluid, ρ =  993.37 kg/m^3^, μ  =  0.000692 kg/m s). To reduce computational load, only half of the chip was modelled due to channel symmetry along the midline. Mesh independence study was conducted. The optimized meshes for the 3D stenosis chip with 0%, 50%, and 80% of constrictions consisted of 2160, 86 678, and 137 892 cells, respectively.

### Whole blood testing

∼3 ml of blood was collected in Sodium Citrate Vacutainer^®^ (BD Biosciences) via venipuncture and used on the same day for microfluidics experiments. Sodium citrate is a suitable anti-coagulant for our study, due to its reported ability to preserve leukocyte-platelet adhesion (by partial Ca^2+^ quenching) with minimal leukocyte activation.^44,45^ Whole blood was stained with Rhodamine-6G (R6G, 1 *μ*g/ml; Sigma-Aldrich) for 30 min at room temperature to enable visualization of leukocytes and platelets adhesion on healthy and TNF-α-treated (10 ng/ml, Peprotech) endothelium (24 h) during perfusion. The microfluidic chip was maintained in the stage-top incubator and perfusion was performed using a peristaltic pump (P720, Instech Laboratories). Prior to each experiment, the flow velocity of the pump was calibrated according to manufacturer's specifications and set to perfuse at desired flow rates. Leukocyte rolling velocities were calculated using ImageJ, from time-lapse images taken over a period of 1 min (0.5 s interval). Neutrophils and monocytes were distinguished using allophycocyanin (APC)-labelled anti-human CD66b (Biolegend, 5 *μ*g/ml) and FITC-labelled anti-human CD14 (Biolegend, 5 *μ*g/ml), respectively, stained for 30 min at 37 °C after blood perfusion and washing steps. Cell counting was performed using ImageJ. To inflame the blood, TNF-α (10 ng/ml) was added to whole blood 2 h prior experiments.

### Study approval

For all subjects, informed consent was obtained. All protocols were approved by Nanyang Technological University institutional review board (IRB-2014‐04-27).

### Statistical analysis

All numerical data were expressed as mean ± standard deviation (s.d.) unless specified otherwise. Mann-Whitney test was used to assess the statistical significance between two groups, and *P *< 0.05 was considered as significant difference.

## SUPPLEMENTARY MATERIAL

See supplementary material for more simulation and experimental figures.
